# Multi-source dataset for urban computing in a Smart City

**DOI:** 10.1016/j.dib.2018.09.113

**Published:** 2018-10-03

**Authors:** Ali Reza Honarvar, Ashkan Sami

**Affiliations:** Computer Science and Engineering and Information Technology Department, School of Electrical Engineering and Computer, Shiraz University, Shiraz, Iran

**Keywords:** Smart City, Internet of Things, Big data, Urban computing

## Abstract

It is vital to capture and analyze, from various sources in smart cities, the data that are beneficial in urban planning and decision making for governments and individuals. Urban policy makers can find a suitable solution for urban development by using the opportunities and capacities of big data, and by combining different heterogeneous data resources in smart cities. This paper presents data related to urban computing with an aim of assessing the knowledge that can be obtained through integration of multiple independent data sources in Smart Cities. The data contains multiple sources in the city of Aarhus, Denmark from August 1, 2014 to September 30, 2014. The sources include land use, waterways, water barriers, buildings, roads, amenities, POI, weather, traffic, pollution, and parking lot data. The published data in this paper is an extended version of the City Pulse project data to which additional data sources collected from online sources have been added.

## Specifications table

**Table:** 

Subject area	*Urban computing*
More specific subject area	*Smart City and Internet of Things*
Type of data	*CSV*
How data was acquired	*Raw data collected from online sources and City Pulse project*
Data format	*Raw and classified*
Experimental factors	–
Experimental features	*Weather, traffic, pollution, parking, land use, waterways, water barriers, buildings, roads, amenities, and POI*
Data source location	*Aarhus, Denmark*
Data accessibility	*The data are available within this paper*

## Value of the data

• This is the first multiple source dataset describing the characteristics of a Smart City from various perspectives to date.
• researchers can use the data to investigate the effects of spatial features on various events in Smart Cities.
• It can be used to identify novel knowledge for urban planning and decision-making through multiple-source urban data integration.
• This data can provide a significant contribution to the literature on urban computing considering various static and dynamic data sources in Smart Cities.

## 1 Data

**Fig. 1:**
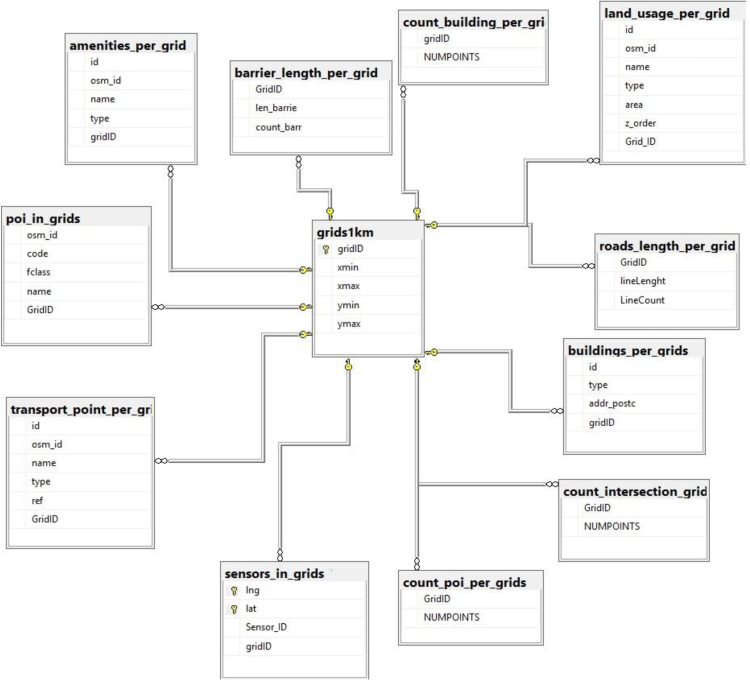
The association and structure of some data sources.

This paper presents data related to urban computing with an aim of assessing the knowledge that can be obtained through integration of multiple independent data sources in Smart Cities. The data covers multiple sources in the city of Aarhus, Denmark. The sources are divided into two categories: static and dynamic. Static data sources present data related to land use, waterways, water barriers, buildings, roads, amenities, and POI. Dynamic data sources present weather, traffic, pollution, and parking lot data during the intended period. The association and structure of some data sources of the data are illustrated in Fig. 1.

The published data in this paper is an extended version of the City Pulse project data [1] to which additional data sources collected from online sources have been added. Meteorological data were acquired from Wunderground׳s website.^1^ Moreover, the concentration of various pollutants as well as road traffic data were taken from the Smart City Pulse project. In addition, the POI data of the city were extracted using Google Maps.^2^ Moreover, the main structure of road networks was obtained from Openstreetmap׳s website.^3^ The data can be used to identify new knowledge for urban planning and decision-making through multiple-source urban data integration. This data can provide a significant contribution to the literature on urban computing considering various static and dynamic data sources in Smart Cities. Some works that have used parts of the City Pulse EU project published data in previous researches are as follows: Maio et al. [2] considers road traffic, parking, and cultural events datasets to evaluate the implementation and deployment of a fuzzy and temporal extension of formal concept analysis for mining high-level semantics from low-level activities. Rathore et al. [3] analyzes the traffic data of the dataset using the proposed big data architecture for a Smart City. Meiyi et al. [4] emulates eight services on the Aarhus datasets to analyze the frequency of conflicts happening across services in a Smart City. Rathore el al [5] proposed smart and intelligent transportation system based on real-time traffic circumstances using graphs. The system was partially evaluated with the traffic data section of the intended dataset.

## 2 Experimental design, materials and methods

### 2.1 Description of study area

**Fig. 2:**
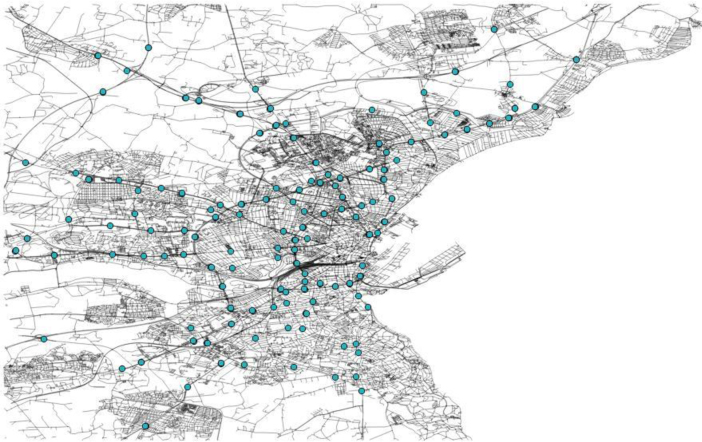
The study area.

The study period was from August 1, 2014 to September 30, 2014, and took place at Aarhus, Denmark. Aarhus covers an area of 91 square kilometers and has a population of approximately 270,000 inhabitants. Some data sources, such as weather, land use, waterways, water barriers, buildings, roads, amenities, and POI were extracted, collected, and preprocessed from online sources. Moreover, the concentration of various pollutants, parking lots, as well as road traffic data, were taken from the City Pulse project using 217 sensors located in the city. Fig. 2 portrays the study area. The green marks on the map represent the main air monitoring stations.

### 2.2 Experimental design

**Fig. 3:**
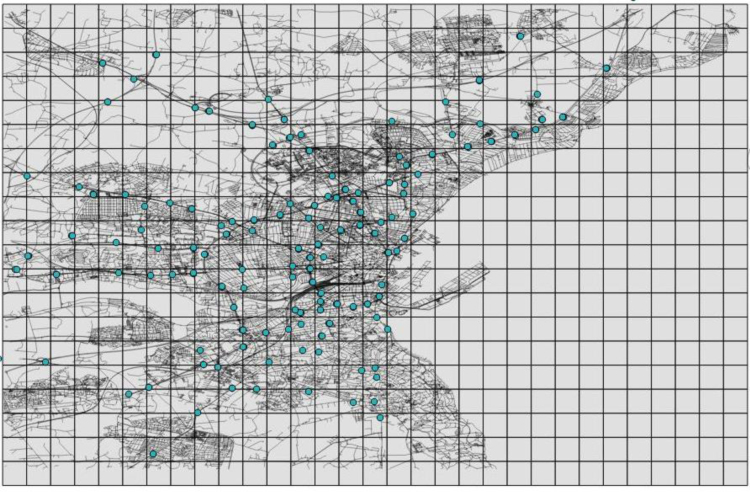
The grids that cover Aarhus.

**Fig. 4:**
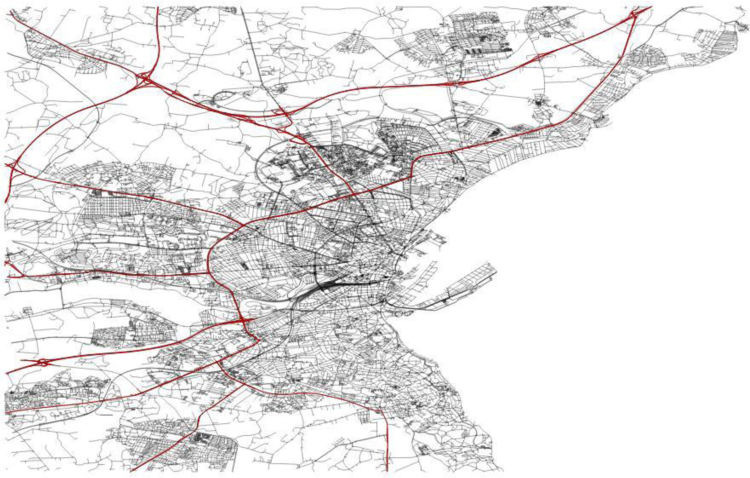
Regions of Aarhus based on main roads, surround by red lines.

To study and evaluate the system performance most researches related to urban computing applied a map splitting method and partitioned the evaluating data into segments according to fixed size grids or variable size regions. For the aim of this study, we follow such methods and published the data in this way. Therefore, the city is divided into segments according to one square meter grids and major roads. When the city is divided into one square meter grids, there are 620 grids that cover the whole city. Fig. 3 illustrates the grids that cover the city. Eleven regions are provided when the urban area of the city is partitioned using the city road network. Fig. 4 shows that Aarhus was divided into 11 regions, based on its major roads. Typically, in a geographical information system (GIS), road networks are divided into seven categories: residential, unclassified, tertiary, secondary, primary, trunk, and motorway. In this paper, the urban division was accomplished according to the main roads, including trunk, primary, secondary, and motorway routes. The supplementary data of this paper was published according to grid partitioning and regions based on the major roads in order to provide a necessary platform for analyzing of Smart City data using multiple data sources.

## Footnotes

1 https://www.wunderground.com.

2 https://developers.google.com/maps.

3 https://www.openstreetmap.org.
